# Physical capability in a rural birth cohort at the age of 52: association with early environmental, nutritional, and developmental factors

**DOI:** 10.1186/s12877-022-02801-5

**Published:** 2022-02-10

**Authors:** Pedro Arroyo, Marcelino Esparza-Aguilar, Verónica Martín-Martín, Juan Carlos Gomez-Verjan, Lorena Parra-Rodríguez, Cinthya Cadena-Trejo, Cecilia Salazar-Pérez, Luis Miguel Gutiérrez-Robledo

**Affiliations:** 1 Department of Clinical Epidemiology, Direction of Research, Instituto Nacional de Geriatría, Blvd. Adolfo Ruiz Cortines No. 2767, Col. San Jerónimo Lídice, Alcaldía La Magdalena Contreras. Distrito Federal, CP. 10200 Ciudad de México, México; 2 Research Unit of Epidemiology, Direction of Research, Instituto Nacional de Pediatría, Insurgentes Sur 3700, Letra C, Alcaldía Coyoacán, C.P. 04530 Ciudad de México, México; 3 Research Unit of Epidemiology, Instituto Nacional de Pediatría, Insurgentes Sur 3700, Letra C, Alcaldía Coyoacán, C.P. 04530 Ciudad de México, México; 4 Dirección de Investigación, Instituto Nacional de Geriatría, Col. San Jerónimo Lídice, Alcaldía La Magdalena Contreras. Distrito Federal, Blvd. Adolfo Ruiz Cortines No. 2767, CP. 10200 Ciudad de México, México; 5 Department of Biomedical Engineering and Gerontechnology. Direction of Research, Instituto Nacional de Geriatría, Blvd. Adolfo Ruiz Cortines No. 2767, Col. San Jerónimo Lídice, Alcaldía La Magdalena Contreras. Distrito Federal, CP. 10200 Ciudad de México, México; 6 Clinical Laboratory, Instituto Nacional de Pediatría. Laboratory of Clinical Chemistry, Insurgentes Sur 3700, Letra C, Alcaldía Coyoacán, C.P. 04530 Ciudad de México, México; 7 Instituto Nacional de Geriatría, Blvd. Adolfo Ruiz Cortines No. 2767, Col. San Jerónimo Lídice, Alcaldía La Magdalena Contreras. Distrito Federal, CP. 10200 Ciudad de México, México

**Keywords:** Developmental origins, Maternal malnutrition, Infant malnutrition, Adult physical capability, Accelerated ageing

## Abstract

**Introduction:**

Midlife physical capability (PC) is associated with developmental factors in the populations of economically developed countries. As far as we know, there is no information for rural populations of low- and middle-income countries. The aim of the study was to investigate the influence of pre- and postnatal factors on midlife objective measures of PC in a 1966–67 birth cohort from a Mexican rural community. The hypothesis was that adverse developmental conditions are associated with low midlife PC.

**Methods:**

In 1966–67, a birth cohort of all children from a poor Mexican rural community was assembled. Data on family socioeconomic status (SES), parental health and nutritional status, birth weight, postnatal growth and feeding patterns were registered. In 2018, out of the 336 cohort members, 118 were living in the community, and eighty-two of them underwent a comprehensive clinical evaluation. The evaluation included grip strength, gait velocity and chair-stand PC tests. In multivariable linear models, PC tests were the dependent variables, and prenatal, birth and postnatal factors were the independent variables. Adjustment for confounding was made with adult anthropometric, body composition, clinical and ageing status variables.

**Results:**

Independent of adult health status and other ageing indicators, lower PC was associated with family organization and SES, parental nutritional status, birth weight, infant postnatal growth velocity, and weaning time. These results indicate that adverse family and environmental conditions that are prevalent in poor rural communities are associated with low midlife PC.

## Introduction

Several investigations have confirmed that exposure to adverse environmental conditions during periods of growth and development is related to adult chronic pathological conditions and age-associated diseases. One of the main identified factors is maternal undernutrition [1]. This association has been explained by the concept of "developmental programming by early-life undernutrition" [2]. The evidence of this relationship comes from studies of populations exposed to prenatal famine episodes and adult health outcomes [3, 4]. However, poor rural communities of low-income and middle-income countries present a different nutritional epidemiological pattern characterized by chronic protein energy maternal and infant undernutrition [5]. Long-standing cohort studies in these types of communities have confirmed the association of maternal and child undernutrition with adult outcomes such as height, education level, income level, birth weight of offspring, BMI, glucose concentration, and blood pressure [6].

We lack knowledge of the association of adverse developmental conditions of subjects born in poor rural communities and the level of physical capability in adulthood. In economically developed countries, it has been found that prenatal and childhood growth are associated with the level of physical capability [7]. The hypothesis of the study was that adverse early developmental conditions accelerate the loss of physical capability in adulthood. Thus, the aim of the study was to assess the physical capability of members of a birth cohort of all children born in a poor rural community of Mexico in 1966–67 and its association with early developmental conditions.

## Methods

### The initial study

The study used data from the Tlaltizapán Birth Cohort, which consisted of all born -336 children- in the rural community of Tlaltizapán, in the State of Morelos, Mexico, between March 1^st^, 1966, and February 28^th^, 1967. The objective of that study was to investigate the impact of childhood malnutrition on growth and mental development [8]. All pregnant women in the community were identified, and data on the household physical and sanitary conditions, and the parents’ height, weight, personal hygiene, formal education, income, and access to mass media were registered. At delivery, the health status and anthropometric measurements of the infant were obtained. The children’s physical and mental development were followed for approximately 14 years [9–12].

### The present study

At the time of the death of the principal investigator in 1998, [13] most of the research files were lost. Twenty years later, we were able to recover information that allowed us to trace the members of the birth cohort and investigate their status. We found that four members died in utero, 22 died in the 1^st^ year (death rate of 65.5/1000 LB) and 6 died before five years (death rate of 17.8/1000 people). It was not possible to locate 133 of the subjects (39.6%); from the remaining subjects, 10 (3.0%) lived in other Mexican States, and 43 (12.8%) lived in the USA. We contacted 118 subjects (35.1%) still living in the community; eighty-two of them (50 women and 32 men), aged 51–52 years, agreed to participate in the study and gave written informed consent to be clinically evaluated. We recovered original information on the household´s sanitary level, family organization, size, and SES, the anthropometric data of both parents, and the mother´s reproductive history. Data on infant birth weight was recovered in 21% of the cases, increments of weight, height, and head circumference in the first six months were recovered in 56.1% of the cases and weaning age was recovered in 46% of the cases.

The clinical evaluation took place at the National Institute of Geriatrics, Mexico City (INGER) between August 2018 and March 2019. The following parameters were evaluated: 1. Anthropometric data: weight, height, arm, waist, and calf circumferences; 2. Body composition: a) Lean, bone and fat mass measured with dual X-ray absorptiometry [DEXA, (*HOLOGIC®, Discovery QDR Series®)], [14] and b) muscular and fat mass with bioimpedance (seca MBCA 514n) [15]; 3. Geriatric syndromes using the a) Cognitive Scale (Mini mental state examination, MMSE®), [16] b) Depression Scale, (CESD-7), [17] c) Risk and Fear of Falls Scales, [18, 19] d) Drug Consumption Scale, [20] e) Fried Frailty Scale, [21] f) Barthel and Lawton Functionality and Daily Life Activities scale, [22, 23] and g) Champ´s Physical Activity and Energy Consumption assessment; [24] 4. PC evaluation: a) Hand Grip Strength (HGS) (Handgrip Dynamometer® with duplicate measurements for each hand, with the subjects in a standing position with the elbow at an angle of 180 degrees, and highest measurement was taken as the final value), [25] b) Gait Speed (GS) (Walkway measuring equipment (Gaitrite® Mod. Platinum 20), [26] c) the 30-s Chair Stand Test (ChS) (Silverfit®), [27] and d) the Standing Balance- Eyes Opened Test (SB) comprised tandem, semitandem, side-by-side stands, and one-legged balance; [28] 5. Self-reported smoking status and alcohol intake, and chronic morbidities; [29] 6. History of heart attack, stroke, cancer, diabetes mellitus, and arterial hypertension; 7. Sitting and standing blood pressure, pulse and respiratory rates, and oxygen saturation were measured in duplicate and with standardized measurements; 8. Biochemical, chemical, and haematological measurements of 36 analytes performed with standardized automatic equipment; and 9. Diagnoses of obesity, osteoporosis, diabetes mellitus, dyslipidaemias, hypertension, and anaemia, with international criteria adapted to the Mexican population. All evaluations were made by standardized professional nurses with the subjects wearing light clothing.

All participants provided written informed consent for the study, genetic data and personal information security. Enrolment and consent procedures for this study were approved by the institutional review boards of the Bioethics and Research of the National Institute of Geriatrics and of the National Institute of Paediatrics under the number INP-INGER 06/2018. All methods employed in the study were performed in accordance with the relevant international guidelines and regulations, as well as those from the Ley General de Salud of Mexico.

### Statistical analysis

We used multiple linear regression in R software (R 3.4.3, Vienna, Austria: R Foundation for Statistical Computing) [30]. The first step of model building aimed to identify the adult clinical variables significantly associated with HGS, GS, ChS scores, and SB scores. Since the SB scores showed low variability, they were eliminated from further analysis. In the second step of the analysis, to control for confounding factors, we built a model for each prenatal, birth and postnatal infant variable, adjusting for adult variables that proved to be significantly associated with the PC variables.

## Results

Table 1 presents the descriptive statistics of the anthropometric, clinical, functional and pathological variables of the participants aged 51–52 years that showed statistical significance in the multivariable models.

**Table 1:** Participants’ somatometric, clinical, functional, and pathological characteristics measured at age 51–52 by sex

**Variable**	**Women (*N* = 50)**				**Men (*N* = 32)**			
**Quantitative variables**	**Median**	**Min**		**Max**	**Median**	**Min**		**Max**
Adult height (m)	1.54	1.40		1.62	1.65	1.53		1.82
Right Leg Length (cm)	82.0	71.0		87.6	84.6	73.0		96.2
Knee height (cm)	42.9	37.0		50.5	46.8	40.3		51.0
Foot width (cm)	9.0	8.0		10.5	10.0	9.0		11.0
Body Mass Index (kg/m^2^)	29.8	20.8		42.1	27.8	21.5		39.6
Lean mass index (muscular kg/m^2^)	15.55	12.30		19.30	18.45	14.90		22.70
Energy expenditure (kcal/day)	2,055.3	1,781.7		2,626.4	2,938.60	2,503.8		4,031.5
Bioelectric impedance phase angle (°)	5.3	3.4		6.4	5.8	4.8		6.6
Resting respiratory rate (breaths/minute)	18	15		24	20	14		25
Resting Diastolic Blood Pressure (mmHg)	70	50		98	77	60		96
Pulse pressure (mmHg)	40	25		80	40	24		68
Uric acid blood concentration (mg/dl)	4.8	3.0		8.2	6.2	4.1		9.0
Depression Score	5	0		19	1	0		12
Frailty index	1	0		4	1	1		3
Grip strength (kg)	22	12		32	40	21		52
Gait speed (cm/s)	105.8	66.1		157.9	107.7	79.3		152.9
Chair stand test (stands/30 s)	16	12		21	17	12		26
Bipodal balance (s)	10	10		10	10	10		10
Semitandem balance (s)	10	10		10	10	10		10
Tandem balance	10	7		10	10	10		10
One-legged balance	10	0		10	10	9		10
	**Women**				**Men**			
**Dichotomous variables**	**n**		**%**		**n**		**%**	
Type 2 diabetes mellitus	10		20%		5		16%	
Systemic arterial hypertension	11		22%		7		22%	
Mini-Mental State Examination (deficit defined by Folstein criterion)	5		10%		4		13%	

Both men and women had near low median height, with low minimum values, and median BMI in the normal range. The median HGS of women was very low; the prevalence of diabetes mellitus and systemic arterial hypertension were high, and there was a deficit in the MMSE test.

Table 2 shows the descriptive statistics of the prenatal, birth, and postnatal variables of the participants, their parents, and family.

**Table 2:** Characteristics of the prenatal, birth and postnatal variables of the participants, their parents, and their family

**Quantitative variables**		**Women**				**Men**			
		**N**	**Median**	**Min**	**Max**	**N**	**Median**	**Min**	**Max**
Participant’s Z score of weight for age at birth		17	-0.68	-2.25	1.26	12	-0.69	-1.32	1.15
Participant’s Z score of head circumference at birth		4	-0.53	-0.75	-0.32	6	0.42	-1.15	1.21
Participant’s increase in arm circumference from the 3rd to 4th month (cm)		46	0.6	-1.3	2.0	29	0.5	-0.9	2.0
Participant’s weaning age (months)		38	16.5	2	28	23	15	1	23
Postpartum maternal BMI (kg/m^2^)		48	23.1	18.0	31.1	32	23.9	19.4	36.6
Father’s BMI (kg/m^2^)		45	21.9	18.7	31.7	31	21.8	14.7	29.5
Father’s formal education (years)		46	2	0	16	31	2	0	11
Number of family members at birth		47	7	3	13	32	5	3	13
Family SES (The higher the value, the lower the status)		45	14	5	19	28	13	7	20
**Ordinal/Dichotomous variables**		**Women**				**Men**			
		**n**	**%**			**n**	**%**		
Participant’s Z score of weight increase from birth to 6 months	<-1	19	41.3%			10	34.5%		
	-1 to +1	20	43.5%			15	51.7%		
	>+1	7	15.2%			4	13.8%		
Participant’s Z score of head circumference increase from birth to 6 months	<-2	6	13.0%			3	10.3%		
	-2 to +1	37	80.4%			16	55.2%		
	>+1	3	6.5%			10	34.5%		
Mother’s height Z score	<-1.5	39	81.3%			26	81.3%		
	-1.5 to 0	9	18.7%			5	15.6%		
	>0	0	0%			1	3.1%		
Mother’s postpartum BMI	<19 kg/m2	1	2.1%			0	0%		
	19 to <25 kg/m^2^	35	72.9%			20	62.5%		
	≥25 kg/m^2^	12	25.0%			12	37.5%		
Father’s BMI	<19 kg/m^2^	1	2%			2	6%		
	19 to <25 kg/m^2^	36	80%			25	81%		
	≥25 kg/m^2^	8	18%			4	16%		
Family’s SES	High	10	22.2%			6	21.4%		
	Middle	21	46.7%			15	53.6%		
	Low	14	31.1%			7	25.0%		
Family type	Nuclear	41	85.4%			23	90.6%		
	Extended	7	14.6%			9	9.4%		

The described variables were those with a significant association with PC outcome variables in the multivariable models. At the participants’ births, the families were large, with a low to middle SES, and a father with a low level of formal education. The median paternal BMI fell within the normal range; based on a postpartum weight measurement, the median maternal BMI presented a distribution from a low (18.0) to a relatively high value (31.1), with a median within the normal range. The categorical mothers’ height distribution showed that they were of short stature compared to the international standard. The median Z score values of birth weight, head circumference, and the increments during the first six months of life showed distributions skewed to low values relative to the international standards [31, 32].

Table 3 presents the HGS, GS and ChS predictors of adult age in the multivariable model.

**Table 3:** Hand grip strength (HGS), gait speed (GS) and chair stand test (ChS) adulthood predictors

**Dependent**	**Predictor**	**Coefficient (95% CI)**	***P ***	**Adjusted*r*2**	**Global*p*-value**
HGS (kg)	(Intercept)	-93.06 (-116.5 to -69.6)	< 0.001	0.8794	< 0.001
	Sex (Female 1, Male 2)	7.93 (4.64 to 11.21)	< 0.001		
	Adult height (m)	64.33 (49.69 to 78.96)	< 0.001		
	Lean mass index (muscular kg/m^2^)	0.83 (0.26 to 1.39)	0.0046		
	Energy expenditure (kcal/day)	-0.01 (-0.01 to -0.001)	0.0092		
	Bioelectric impedance phase angle (°)	2.13 (0.54 to 3.72)	0.0094		
	Pulse pressure (mmHg)	-0.1 (-0.18 to -0.02)	0.0207		
	Depression Score	-0.15 (-0.3 to < 0.0001)	0.0501		
GS (cm/s)	(Intercept)	212.7 (127.29 to 298.12)	< 0.001	0.462	< 0.0001
	Sex (Female 1, Male 2)	-22.05 (-35.51 to -8.58)	0.002		
	Body Mass Index (kg/m^2^)	-2.08 (-2.91 to -1.26)	< 0.001		
	Grip strength (kg)	1.32 (0.69 to 1.96)	< 0.001		
	Right Leg Length (cm)	-1.78 (-2.76 to -0.79)	< 0.001		
	Knee height (cm)	-1.56 (-2.89 to -0.24)	0.021		
	Foot width (cm)	7.17 (0.9 to 13.43)	0.026		
	Resting respiratory rate (breaths/minute)	1.78 (0.47 to 3.08)	0.008		
	Resting Diastolic Blood Pressure (mmHg)	0.56 (0.17 to 0.96)	0.006		
	Uric acid blood concentration (mg/dl)	4.31 (1.79 to 6.82)	0.001		
	Type 2 Diabetes mellitus (with diagnosis = 1)	11.1 (2.48 to 19.71)	0.012		
	Systemic Arterial Hypertension (with diagnosis = 1)	-9.85 (-18.26 to -1.44)	0.022		
	Mini-Mental State Examination (Normal = 0, Deficit = 1)	-11.19 (-21.19 to -1.19)	0.029		
ChS (stands/30 s)	(Intercept)	38.56 (22.74 to 54.38)	< 0.0001	0.1342	0.0027
	Sex (Female 1, Male 2)	3.18 (1.39 to 4.96)	0.0007		
	Adult height (m)	-15.82 (-26.59 to -5.05)	0.0045		
	Frailty index	-1.14 (-2.02 to -0.26)	0.0111		

Related to HGS, male sex, height, lean mass, and phase angle (bioimpedance indicator of muscular mass) had significant positive effects, while energy expenditure and pulse pressure had negative effects; the depression score showed a trend towards significance with a negative effect. Regarding GS, foot width, resting respiratory rate, diastolic blood pressure, uric acid blood concentration, and the diagnosis of diabetes mellitus showed a positive association. Variables negatively associated with GS were male sex, BMI, leg length, knee height, the diagnosis of hypertension and poor performance on the MMSE**.** With the ChS test, male sex was the only variable that was positively associated, while height and the frailty index variables were negatively associated.

Table 4 shows the prenatal, birth and postnatal variables with significant effects on HGS, GS, and ChS scores, each of which was adjusted by the adult predictors described in Table 3.

**Table 4:** Effects of prenatal and childhood variables on physical capability tests adjusted by adulthood variables

	**Variable**			**Coefficient (95% CI)**	***P***	**Adulthood variables included in the model**	**Adjusted*R*2**	***N***
Grip strength (kg)	Z score of weight for age at birth (S.D.)			4.5 (0.6 to 8.4)	0.027	Phase angle, Depression score	0.4633	29
	Z score of head circumference at birth (S.D.)			2.14 (0.77 to 3.51)	0.012	Sex, energy expenditure, pulse pressure, depression score	0.9916	10
	Prenatal family SES (The higher the value, the lower the status)			-0.44 (-0.75 to -0.14)	0.005	Sex, lean mass index, pulse pressure	0.7894	72
	Post-gestational maternal BMI (kg/m^2^)			0.27 (0.01 to 0.53)	0.040	Sex, adult height, energy expenditure, phase angle, depression score	0.8688	79
	Prenatal paternal BMI at birth (kg/m^2^)			0.31 (0.01 to 0.62)	0.040	Sex, adult height, lean mass index, pulse pressure, energy expenditure	0.8673	75
	Number of family members at birth			0.67 (0.34 to 0.99)	< 0.001	Sex, adult height, lean mass index, pulse pressure, energy expenditure	0.8899	78
	Increase in arm circumference from 3rd to 4th month (cm)			-1.17 (-2.24 to -0.1)	0.033	Sex, height, lean mass index, depression score, pulse pressure, energy expenditure	0.8749	74
	Z score of weight increase from birth to 6 months		-1 Z to < + 1 Z (vs < -1 Z)	-1.67 (-3.49 to 1.49)	0.071	Sex, height, lean mass index, energy expenditure, phase angle, pulse pressure, depression score	0.8867	74
			> + 1 Z (vs < -1 Z)	-2.78 (-5.39 to -1.75)	0.037			
	Z score of head circumference increase from birth to 6 months		< -2 Z (vs -2 Z to + 1 Z)	-0.58 (-2.92 to 1.77)	0.625	Sex, height, lean mass index, energy expenditure, angle phase, pulse pressure, depression score	0.893	74
			> + 1Z (vs -2 Z to + 1 Z)	-3.19 (-5.27 to -1.11)	0.003			
Gait speed (cm/s)	Living in a nuclear (vs extended) family at birth			-11.06 (-21.75 to -0.37)	0.0427	Right leg length, foot width, BMI, diabetes mellitus, MMSE result, blood uric acid	0.2906	80
	Weaning age (months)			0.55 (0.02 to 1.07)	0.0423	Sex, right leg length, knee height, BMI, grip strength, blood uric acid, resting diastolic blood pressure, resting respiratory rate	0.4958	61
	Prenatal father's education level (years)			1.5 (0.43 to 2.57)	0.0067	Sex, right leg length, foot width, BMI, grip strength, resting diastolic blood pressure, blood uric acid, diabetes mellitus, MMSE	0.4902	76
	Post-gestational Mother's BMI	< 19 kg/m^2^ (vs normal)		-32.86 (-62.43 to -3.28)	0.0300	Right leg length, knee height, grip strength, BMI, diabetes mellitus, blood uric acid	0.3968	79
		≥ 25 kg/m^2^ (vs normal)		-8.64 (-15.84 to -1.44)	0.0193			
	Prenatal family's socioeconomic status	Medium (vs High)		-3.09 (-11.76 to 5.58)	0.4786	Sex, right leg length, knee height, foot width, grip strength, BMI, resting diastolic blood pressure, resting respiratory rate	0.4331	72
		Low (vs High)		-11.83 (-21.97 to -1.68)	0.0231			
	Prenatal Mother's Height Z score	< -1.5 Z score (vs ≥ -1.5 to < 0 Z)		-13.78 (-22.87 to -4.69)	0.0035	Right leg length, grip strength, BMI, diabetes mellitus	0.3415	79
		≥ 0 Z score (vs ≥ -1.5 to < 0 Z)		-32.07 (-64.04 to -0.1)	0.0493			
	Head circumference growth velocity from 0 to 6 months age (Z score)	< -2 Z score (vs -2 to < + 1 Z)		-16.75 (-27.32 to -6.18)	0.0023	Right leg length, foot width, BMI, resting diastolic blood pressure, resting respiratory rate	0.3252	75
		≥ + 1 Z score (vs -2 to < + 1 Z)		-13.5 (-22.96 to -4.05)	0.0058			
Chair stand test (stands/30 s)	Weaning age (months)			-0.12 (-0.21 to -0.03)	0.0110	Sex, adult height, frailty index	0.2721	61

^*^Every childhood variable coefficient corresponds to a single and different multivariable model

The higher the body size at birth (birth weight, birth head circumference), family SES, parental nutrition (maternal and paternal BMI), and family size, the higher the adult maximal HGS. Infant growth measurements in the first six months were negatively associated with HGS**:** higher increment values of weight and head circumference, standardized for age and sex, were negatively associated with HGS; increases in arm circumference from the 3^rd^ to 4^th^ months of age also had a negative effect. Performance on the GS test was negatively affected by maternal size (high and low BMI, and low and above-median height), low family SES, living in a nuclear family at birth and both slow and accelerated head circumference growth. Having been weaned later and having a father with more formal education were variables that were associated with better performance on the test. For the ChS test, having a long weaning period was the only variable found to be significantly and negatively associated.

## Discussion

The specificity and strength of the associations of the PC tests with early influences found in the present study can be derived from the set of adjustment variables associated with each of them, i.e., for HGS, the main predictive variables were adjusted by sex, height, and muscular mass; for GS, they included sex and body shape variables, such as leg length, foot width, and BMI; and in the ChS model, the only variables were sex, height and the frailty index. These differences in adjustment variables by test indicate the different physiological parameters required to adjust in modelling the environmental and developmental associations.

Our results confirm the association of prenatal and birth factors with midlife PC in adults. These results are consistent with the hypothesis of the developmental origins of adult health and disease [2, 33, 34]. The negative association of HGS with weight and head circumference increments during the first six months of life that was found provides new insight into early life adverse influences on adult PC in a group of underprivileged subjects with a rural background. In the case of populations of European ancestry, the Northern Finland Birth Cohort Study, an investigation on growth during the first year of life, reported that greater infant weight gain was associated with lower muscle endurance and poorer aerobic fitness in adulthood, although these effects could have been mediated by adult body size [35]. In our case, the adjustment variables included height and muscle mass. Other studies in economically developed populations have shown that unfavourable influences such as low SES, maternal education and income experienced during prenatal and childhood periods were associated with weaker HGS, low muscular strength, or worst PC in adulthood [33, 36–40]. There are also reports of positive effects of birth weight on muscle strength both in association with or independent of body size [41–43].

In our study, the associations of environmental, prenatal, birth and postnatal factors with midlife PC tests were positive or negative. For instance, better midlife HGS performance was positively associated with family SES and size, maternal and paternal nutritional status, and birth weight. These results suggest that the prenatal improvement of these parameters could result in better future ageing trajectories and adult PC performance. On the negative side of the spectrum, postnatal growth velocity indicators showed a negative association with HGS, a result that is consistent with the high incidence of postnatal malnutrition due to diarrhoeal diseases and the late introduction of foods that are different from the mother´s milk, a pattern commonly found in Tlaltizapán [11, 12]. In the same way, in a Finnish cohort study, BMI increases from ages 2 to 11 years in males (but not in females) was positively associated with frailty at 67–79 years of age [44]. In contrast, in a British cohort, height increases, from ages 2 to 7 years, was also related to HGS but with a positive effect [42]. Regarding GS, better performance was associated with higher levels of the father´s formal education, which is an SES indicator. This result is consistent with a Chinese study that reported that an illiterate paternal education level (vs. literate) in early life was associated with an increased risk of frailty at ≥ 60 years of age [45]. Infant feeding habits have been associated with the development of malnutrition in rural populations. In the present study, better GS performance was associated with late weaning, a probable nutritional protective factor in a poor rural environment; the median weaning age of the participants was 15 and 16 months for men and women, respectively, with a range of 1 to 28 months. Sanjur, Cravioto, and coworkers reported the practice of prolonged breastfeeding among the cohort’s mothers [46, 47]. Similarly, the early introduction of foods that were different from breast milk was a risk factor for marasmus because of the deficit of high-quality protein and energy. As was the case for HGS, postnatal growth velocity indicators were negatively associated with GS performance, confirming the influence of postnatal malnutrition on midlife PC. In addition, lower GS performance was associated with social factors such as family type and SES. To interpret these findings, it can be speculated that poor nuclear rural families lack sufficient support from other family members. This hypothesis is consistent with other studies where low SES in childhood or before the age of 16 was associated with low GS in adulthood [37, 48]. Indicators of maternal nutritional level (post-gestational BMI and height) were negatively associated with GS performance. Height is considered a cumulative index of nutrition, [49] and the cohort’s mothers were of short stature; Cravioto et al. reported that the median height of the cohort’s mothers was 147.5 cm, and the first quartile was only 145.5 cm [8], a short stature compared with international standards. The multivariable model for the ChS test showed only a weak negative association with weaning age, a result that contrasts with studies that reported the influence of social variables such as the father’s occupational class and low SES on ChS performance [33, 50].

Overall, our results indicate the negative influence of a poor rural family environment, a low nutritional status of parents, especially of the mother, and low intrauterine and postnatal growth, the latter associated with inadequate infant feeding practices, on midlife PC. At the time of the cohort’s inception, the relatively small Mexican rural community of Tlatizapán had a low standard of living, as documented by Cravioto et al. in the Monograph [8]. The health and nutrition surveys at that time revealed that extended families lived in households with poor sanitary conditions and consumed a monotonous diet limited in energy and high-quality protein. Thus, early deficient nutritional status is most likely a contributing factor to the low average HGS of the participants, compared with the average of adults of the same age of economically developed countries and even of residents of Mexico City, as shown in Table 5 [25, 51–53]. This observation is consistent with the report by Dodds et al. [54], who showed that normative HGS data from developing regions were lower than those of developed regions of the world.

**Table 5:** Handgrip strength by sex in our study and four community studies with a similar age group

Sex	Source	Age range	*N*	Mean	95%CI	Significance*
Females	Current study	51–-52	50	22.2	20.9—23.5	Lowest
	Mexico City [28]	50 – 59	75	25.0	24.1—25.9	2nd Lowest
	Korea [54]	50 – 54	535	26.9	26.5—27.3	
	USA [55]	50 – 59	780	27.5	27.1—27.9	
	UK [56]	51 – 55	4250	27.5	27.3—27.7	
Males	Current study	51–-52	32	38.5	36.1—40.9	Lowest
	Mexico City [28]	50 – 59	74	42.0	40.9—43.1	
	Korea [54]	50 – 54	386	43.3	42.7—43.9	
	USA [55]	50 – 59	721	42.1	41.5—42.7	
	UK [56]	51 – 55	3743	46.2	45.9—46.5	Highest

^*^Significant difference if 95%CI of one group does not imbricate with 95%CI of another

Muscular strength, an important component of PC, particularly HGS, is a function of muscle mass development during the foetal stage. In a Finnish study of elderly frail women, postpartum maternal BMI was related to muscle strength in specific (but not in most) muscle groups: the daughters of obese mothers were weaker than the daughters of lean mothers [57]. On the other side of the nutritional spectrum, it has been demonstrated that maternal undernutrition limits the proliferation of precursor muscular cells and reduces the number of fibres formed, a process thought to be mediated by epigenetic changes that could explain, among other factors, the lasting effects on midlife PC [56, 58]. At the time the original research was implemented in Tlaltizapán, nearly 70% of the Mexican population lived in rural communities of similar conditions, which favoured the incidence and prevalence of malnutrition. The extensive rural–urban migration that has taken place in Mexico in recent decades has reversed that proportion. The present urban population faces a high prevalence of obesity, diabetes, and hypertension. Demographic transitions in low-income countries has been characterized by four intergenerational cycles [57]. In our study, we documented low nutritional status in at least three generations: the cohort’s grandmothers (data not shown), and stunted mothers giving birth to undernourished children who, in midlife, suffer from chronic conditions such as obesity, diabetes and hypertension.

The loss of information from the original study and the reduced sample size, partially due to refusal of the clinical evaluation, are the main limitations of the study. However, our study, although on a limited scale, contributes to the understanding of the present health problems and future ageing trajectories that populations, such as the Mexican population, are likely to face in the future.

## Conclusions

The midlife physical capability of adults born in a rural community with low environmental and socioeconomic conditions is associated with low parental nutritional status, intrauterine and postnatal growth retardation and late weaning. Physical capability in adulthood is an indicator of pathological ageing trajectories. Our results stress the importance of promoting health conditions during development as a means to prevent future pathological ageing trajectories.

## Data Availability

The data sets generated and/or analyzed during the current study are not publicly available due to patient privacy, but are available from the corresponding author on reasonable request.

## Abbreviations

PC: Physical capability
LB: Live births
SES: Socioeconomic status
HGS: Hand grip strength
GS: Gait speed
ChS: Chair stand test
SB: Standing balance
BMI: Body mass index
